# Prevalence of work-related burnout and associated factors among police officers in central Gondar zone, Northwest Ethiopia, 2023

**DOI:** 10.3389/fpubh.2024.1355625

**Published:** 2024-04-23

**Authors:** Anmut Endalkachew Bezie, Dawit Getachew Yenealem, Azanaw Asega Belay, Alebachew Bitew Abie, Tadiwos Abebaw, Christian Melaku, Yimer Mamaye, Amensisa Hailu Tesfaye

**Affiliations:** ^1^Department of Occupational Health and Safety, College of Medicine and Health Sciences, Wollo University, Dessie, Ethiopia; ^2^Department of Environmental and Occupational Health and Safety, College of Medicine and Health Sciences, Institute of Public Health, University of Gondar, Gondar, Ethiopia; ^3^Department of Environmental Health, College of Medicine and Health Sciences, Wollo University, Dessie, Ethiopia

**Keywords:** Copenhagen Burnout Inventory, work-related burnout, police officer, prevalence, psychosocial risk factors, burnout, Ethiopia

## Abstract

**Introduction:**

Work-related burnout is a state of physical and psychological fatigue and exhaustion resulting from chronic workplace stress related to work. The police workforce is vulnerable to this psychosocial hazard, which affects service delivery by police workers. However, there is little evidence about the prevalence of work-related burnout and associated factors among police officers in Ethiopia. Therefore, this research investigated the prevalence and predictor variables of work-related burnout among police officers in central Gondar zone, Northwest Ethiopia, 2023.

**Methods:**

An institution-based cross-sectional study was carried out from April 12 to May 12, 2023. A sample of 633 police officers was recruited through multistage random sampling techniques. To measure work-related burnout, a standardized, self-administered Copenhagen Burnout Inventory was used. To enter the collected data, EpiData V 4.6 and to analyze SPSS V 26 were used. To examine the association between work-related burnout and its predictor variables, both bivariable (*p* < 0.2) and multivariable (<0.05) logistic regression analyses were performed, and statistical significance was established via multivariable logistic regression.

**Results:**

The response rate in this study was 96.05% (*n* = 608). The majority, 452 (74.3%) of the police officers, were male; the median (IQR) age of participants was 28. In the past six months, 45.7% of the police officers had experienced work-related burnout (*n* = 278). Being female, having a high job demand, having a high level of organizational police stress, having a moderate level of operational police stress, having a high level of operational police stress, experiencing job dissatisfaction, and sleeping troubles were risk factors significantly related to the occurrence of work-related burnout among police officers.

**Conclusion:**

According to this study, a significant number of police officers suffer from burnout due to their work. Police officers’ work-related burnout was found to be influenced by factors such as sex, job demands, job satisfaction, workplace stress, organizational police stress, and sleeping troubles. To address this problem, improving the handling of work pressure, developing a stress management program, finding joy at work by improving interpersonal relationships and working conditions, offering support and inspiration to female police officers, and ensuring sufficient and restful sleep are advised.

## Introduction

Work-related burnout (WRB) is a condition of both physical and psychological fatigue and exhaustion that arises due to the inability to effectively cope with chronic stress in the workplace (1). It is a gradual decline in enthusiasm, motivation, vitality, and empathy due to the demands of the job (2). It is distinguished by feelings of fatigue, a greater mental distance from one’s work, feelings of cynicism about one’s employment, and a decline in professional competence (3). According to the 11th International Classification of Diseases (ICD-11), burnout is considered an “occupational phenomenon” by the World Health Organization (WHO) (4). WRB is a prominent occupational hazard of the twenty-first century, and it is presently attaining epidemic proportions in the realm of employment, impacting worker efficiency and diminishing organizational effectiveness (5).

Police work is mandatory for the development of a country since national development might not be feasible if a nation’s peace and security are at stake, and without it, there is a threat due to civil unrest and insecurity. Police officers therefore play a crucial role in upholding public order, combating crime, and enforcing the law (6). There are numerous professional challenges that make police work vulnerable to work-related burnout. Their job is one of the most demanding and stressful jobs in the world (7), characterized by long working hours, demanding schedules, irregular shift work, and frequent night shifts, which revealed burnout (8). In addition, on a daily basis, they face challenging and potentially dangerous situations, such as the occurrence of murder, and violent crimes are frequent. They also regularly encounter distressing situations and stressors associated with managing and organizing their work, and these demanding job requirements and responsibilities might lead to more time constraints and less flexibility in how the work is done, which leads to psychological strain and burnout (9, 10). When comparing occupational stress among different professions, police work was the second-most stressful and challenging job (11), with a wide range of operational and work-related stressors, such as face-to-face violence, negative interactions with citizens (12), continued critical life-threatening situations, and injuries that put them at risk for burnout. This stress, in turn, adversely affects their mental and physical well-being, performance, and interactions with the public. Mental health problems become rampant due to the increasing prevalence of precursors, including burnout, depression, and anxiety among police officers (13). Police officers had a higher suicide rate than the whole working population as a result of burnout at work (14). Work-related burnout is the most common cause of reduced productivity, absenteeism, suicidal ideation (15), worse job performance, increased turnover (16), cynicism, fatigue, aggression, violence (11), alcohol or substance misuse (17), and physical health issues (e.g., high blood pressure, ischemic heart disease, poor sleep, cardiovascular disease, obesity, etc., compared with the general population) (18).

The peculiar nature of policing increasingly becomes a reason for the ever-growing burden of work-related burnout around the world (19). In the United States, 19% were emotionally exhausted, and 13% experienced severe depersonalization (20), Spain (32.2%) (21), Sweden (28%) (22), and Chile (28%) (23). In a developing country, Bulgaria (74.53%) (24); Mexico (31.1%) (25); South Africa, where a high prevalence of exhaustion and cynicism was due to passive coping strategies (26); and Kenya (30.9%) (27). The occurrence of WRB among police officers is multifactorial: inadequate salary and unpaid overtime; excessive workloads (28); work–family conflicts (29); overburdened administrative roles; policy changes; and staff shortages (30).

Previous research has shown that sex (17, 31, 32), age (33, 34), years of experience (21, 35, 36), educational level (37), marital status and family size (21), monthly salary (38), organizational rank, and working position (39) all play a role in burnout. For example, the highest stressor that causes burnout in police officers is experienced after six to ten years (36), and accumulated experience leads to wisdom and better coping skills (35); police officers who did not have a partner scored significantly higher on the burnout scale than did those who did; and police officers without children showed a higher level of personal achievement than did those with children (21). Organizational rank and working positions were statistically associated with burnout; that is, the rate of burnout increases with seniority and is greater in day-to-day criminal investigations than in other roles (39). Low salaries were the main predictor of burnout (38). Officers with higher educational levels were less affected by burnout (37). Rural police officers were more likely to experience somatization as compared with their urban counterparts, and the perceived inadequate resources and training may partly explain the rural–urban differences (40). Urban police officers are more likely to have both depression and burnout, and the rise in burnout symptoms explains the depressive symptoms (41). Police officers who smoke, take substances that make them drowsy, or promote sleep usage in an attempt to remain awake, which leads to burnout, increased stress, and errors due to tiredness (42). Physical activity has been demonstrated to be positively and significantly correlated with a lower risk of burnout (17, 43). Several studies have investigated the psychosocial health indicators associated with WRB. For example, officers who worked more extended shifts had a greater chance of experiencing emotional exhaustion (44), and those who worked both day and night shifts more commonly experienced high levels of burnout compared with those who worked only during the daytime (45). The main predictors of burnout are inadequate resources and an overwhelming workload (38), and burnout increases with poor leadership, role conflicts, the fear of violence, and time constraints (39). Workplace control, job demand, and job uncertainty are all associated with burnout (31). Job dissatisfaction was the main predictor of burnout (46). Dealing with family conflicts, abusing children, killing someone while performing their duties, and witnessing the death of a fellow officer are the most commonly reported stressors that cause burnout (47).

Higher levels of social support were protective factors against burnout (48). Excessive demands on individuals’ ability to control, work overload, role conflicts and ambiguity, personal work relationships, lack of recognition, little or no participation in decisions, and job stability uncertainty are critical factors that contribute to work stress and ultimately burnout (25). Organizational factors, including inadequate resources, poor working conditions, feeling unfairly compensated, a lack of opportunities for promotion, and inadequate time with family, are causes of burnout (49). Work-related burnout needs to be addressed immediately, as it is the most significant health issue affecting the service delivery of policing in Ethiopia. However, there is scanty evidence regarding the prevalence of WRB and its predicted variables among police officers. Therefore, this study was carried out to investigate the prevalence and predicted variables of WRB among police officers in central Gondar zone, Northwest Ethiopia. The findings of this research will offer baseline information for designing and promoting wellness programs for specific professionals to minimize and control WRB. In addition, the study’s findings will give pertinent information for stakeholders, police administrators, and legislators to develop and put into practice efficient preventive and control measures.

## Methods and materials

### Study design, period, and setting

An institutional-based cross-sectional study was carried out from April 12 to May 12, 2023. The study was conducted in the central Gondar zone, which is the oldest and most historical place in northwestern Ethiopia. It is located 737 km from Addis Ababa, the capital city of Ethiopia, and 173 km from Bahirdar, the regional capital city of Amhara (50). Based on the information obtained from the central Gondar zone and the Gondar city police administration, the central Gondar zone has 15 woredas and a 4-woreda city administration that puts its main location in Gondar city, 1,272 police officers, 26 police stations, and more than 40 police centers (community policing). Among them, 420 police officers worked in Gondar city, and 852 worked in 15 woredas and 4 woreda city administrations.

### Population and eligibility criteria

The study population consisted of all police officers in the central Gondar zone. Police officers who had at least six months of experience in the selected woredas (an administrative division of Ethiopia, managed by a local government) were eligible for this study (51, 52). Participants who were on annual leave, maternity leave, sick leave, or had an apprentice status during the data collection period were excluded from the study.

### Sample size determination and sampling procedure

The sample size was calculated using a single population proportion formula with the following assumptions: proportion (*p* = 0.5), margin of error (*d* = 5%), and *Z* score ( $Z\alpha {/}_{2}$ = 1.96) corresponding to 95% of the confidence interval.


n=Zα/22p1−pd2,n=1.96^20.51−0.5/0.05^2=384

Considering a 10% nonresponse rate and 1.5 design effects, the total sample size (*n*) was: 384* 0.1 + 384 = 38.4 + 384 = 422, and after multiplication by 1.5, it became 633.

The sample was taken from the central Gondar zone and included a total of 1,272 police officers. The Central Gondar Zone has 420 police officers from Gondar city and 852 police officers from four woreda city administrations and 15 woredas.

By considering Gondar city and four woreda administrations as five woredas, the total number of woredas in the central Gondar zone becomes 20. Since these woredas are dispersed among each other, it is difficult to account for all 20 woredas due to feasibility issues (time, money, transport, and hassles). Therefore, based on WHO sampling assessment standards, 40% of the total woredas became 8 (53). Simple random sampling (lottery method) was used to select eight woredas. To allocate a representative sample in each woreda, a proportional allocation to the size of the population was used. Finally, a simple random sampling method was used to select study participants from each selected woreda.

### Operational definitions and measurement of variables

*Work-related burnout:* was measured using a Copenhagen Burnout Inventory (seven items). The presence or absence of work-related burnout was indicated by an average total score of 50, <50 = 0 (no burnout) and ≥ 50 = 1 (burnout) (1, 54).

*Police officers:* are professionals who focus on keeping or enforcing law and order, investigating crime, and supporting crime prevention (55). The police officers in this research were those who worked for Gondar city and the central Gondar zone police administration and had at least six months of experience.

*Alcohol drinking:* the consumption of any alcohol-based beverage, whether locally or industrially produced, by police officers at least twice per week (56).

*Khat chewing:* A police officer chewed khat three times a week for the last six months prior to the study (57). Khat is a plant whose leaves are chewed and drunk as tea in the East African region, as well as in some parts of the Middle East, for stimulation or enlightenment (58).

*Cigarette smoking:* a police officer who smoked at least one stick of cigarettes per day for the six months prior to the study (59).

*Doing physical exercise:* individuals who perform any kind of physical exercise at least two times per week for 30 min (60).

*Medication use* was assessed using a single, yes-or-no question to gauge participants’ coping strategies for burnout at work (61).

### Psychosocial work factors

*Job stress:* operational and organizational police stress tools were used to assess the job stress of police personnel. A score of less than 2.6 on the organizational police stress scale was considered low stress; a score between 2.7 and 3.9 was considered moderate stress; and a score of 4.0 or higher was considered high stress. According to the Operational Police Stress tool, a score ≤2.0 indicates low stress, a score between 2.1 and 3.4 indicated moderate stress, and a score of ≥3.5 indicates high stress (51).

*Role conflict*: four items; *role clarity and social relations:* three items; and *job security:* two items with a five-point Likert scale and dichotomized by their mean value (62).

*Sleeping trouble* with four items, job recognition with three items (63), and job demand with four items (9) on a five-point Likert scale and dichotomized by their median value.

*Job satisfaction:* Ten items from a generic job satisfaction scale were used to measure job satisfaction; a score of 10–31 indicated satisfaction, while a score of 32 or higher indicated dissatisfaction (64).

*Social support:* Participants answered nine questions concerning the assistance they received from family, coworkers, and superiors. A mean scale score falling between 1 and 2.9 categorized as low social support, a score between 3 and 5 as moderate social support, and a score between 5.1 and 7 as high social support (30, 65).

*Work–family conflicts:* six items on a four-point Likert scale, ranging from 0 never to 4 often, and dichotomized by median value (66). Of the six questions, work–family conflict was addressed in the first four, and family–work conflict was addressed in the final two (66).

### Data collection procedures and tools

Standardized, self-administered Copenhagen Burnout Inventory (CBI) questionnaires were used to collect the data. The survey had four sections. The socio-demographic characteristics such as age, sex, education, work experience, having children, monthly salary, organizational rank, and working position were covered in the first section. The questions in the second category were designed to evaluate data regarding burnout at work. Work-related burnout was measured using the Copenhagen Burnout Inventory, which has been applied and validated in earlier studies (67, 68). Behavioral factors such as alcohol consumption (yes or no), khat chewing (yes or no), cigarette smoking (yes or no), medication use for stress relevance (yes or no), physical exercise (yes or no), and work misconduct issues (yes or no) were included in the third section of the questionnaires. The operational and organizational police stress and Copenhagen Psychosocial Questionnaire are both employed in the fourth item, which assesses numerous working conditions. Operational and organizational police stress were used to assess the job stress of police officers. They are reliable and valid indicators of policing stressors. Each questionnaire had 20 items that were scored from 1 (no stress at all) to 7 (a lot of stress). They are free for non-commercial, educational, and research purposes (51). Participants indicate the degree to which each item contributed to their burnout during the preceding six months. The mean of each scale was used to obtain the summary scores. Copenhagen Psychosocial Questionnaire: one item related to job demand, “Do you have enough time for your work tasks?” was reversed (54). Burnout status was divided into three subscales according to the Copenhagen Burnout Inventory: personal, work-related, and client-related burnout (69, 70). The questionnaire’s three distinct sections were created to be utilized in various contexts. However, we used the WRB domains of the CBI’s in this investigation. The seventh and final item on the list—“Do you have enough energy for family and friends during leisure time?”—was reversed in scoring (69, 70). The reliability and validity of CBI are confirmed by the PUMA project (Burnout, Motivation, and Job Satisfaction). The results showed that the three scales had minimal non-response rates and extremely good internal reliability. This tool has been translated into seven languages, i.e., English, Japanese, Mandarin, Cantonese, Swedish, Finnish, French, and Slovenian, and is currently being used in many nations (1). It is a generic instrument for evaluating workplace risk factors that is free to use. The reliability and validity of CBI were tested in Africa among Nigerian resident doctors and showed the highest Chronbach’s alpha (71). In Ethiopia, this tool is used to assess burnout among midwives and school teachers, with a high reliability (Chronbach’s alpha) of 0.89 (72, 73). The seven items on the list on the CBI were used to measure burnout at work. The internal consistency of the current investigation was 0.86. The items elicited responses ranging from very low/never = 1, low/rarely = 2, somewhat/sometimes = 3, high/often = 4, and very high/always = 5. Each question has a 5-point Likert scale (never, seldom, sometimes, often, and always) response option, which study participants picked from. Before calculating the mean scores for each of the components, the negatively worded questions were reversed where necessary. Weighted scores (percentages) were assigned to each option as follows: never (0), seldom (25), sometimes (50), often (75), and always (100). The scale labels were recorded in accordance with the questionnaire designers’ instructions, using the following formats: 1 = 0, 2 = 25, 3 = 50, 4 = 75, 5 = 100; for reverse coding, 1 = 5, 2 = 4, 3 = 3, and then all the items were summed (70, 74). Measurements and calculations were performed for Chronbach’s alpha reliability test. For instance, organizational police stress 20 items (Chronbach’s alpha = 0.871), operational police stress 20 items (Chronbach’s alpha = 0.911), role clarity three-items (Chronbach’s alpha = 0.74), role conflict four items (Chronbach’s alpha = 0.77), sleeping troubles four items (Chronbach’s alpha = 0.84), job demand four items (Chronbach’s alpha = 0.74), job recognition three items (Chronbach’s alpha = 0.72), social relations three items (Chronbach’s alpha = 0.73), job satisfaction ten items (Chronbach’s alpha = 0.79), social support nine items (Chronbach’s alpha = 0.90), and work–family conflict six items (Chronbach’s alpha = 0.78). Therefore, the above data were collected by considering the police officer’s time, place, and person. In terms of time, study participants were approached by considering their work schedules, mobility at home, field work, and office work. In terms of place, we approach them either individually or in groups at their offices for privacy and honesty in their information. In addition, in terms of personnel, four data collectors and two supervisors were assigned to collect the data.

### Data quality assurance

The questionnaires were originally written in English, translated into Amharic, the native tongue, and then returned to English by the author with the assistance of linguists. Two psychiatric experts who had prior expertise and knowledge of the data collection procedure were hired as supervisors, and four environmental, occupational health, and safety specialists were hired to gather the data. Before beginning the actual data collection, the supervisors and data collectors attended two days of training and orientation covering topics such as informed consent, study goals, confidentiality, and how to ask clear and concise questions. Both lectures and discussions were used to deliver the training. Before the actual data collection, thirty-two samples were pre-tested on the questionnaires, which were excluded from the final analysis. Estimates of the time required for data collection were determined, and based on the pre-test results, some adjustments were made for question misinterpretations. For instance, based on their information, the organizational rank, working position, and educational level were rearranged. Every day, the investigator verified that the questionnaire was accurate and comprehensive.

### Data management and analysis

After the data were verified to be accurate, they were imported into Epi-data version 4.6, exported, cleaned, and analyzed with SPSS version 26. Cross-tabulations were performed to assess the relationship between the explanatory variables and the outcome variable, and frequency distributions and percentages were employed to show descriptive statistics. The variables’ multicollinearity, outliers, and normality were assessed before the bivariable and multivariable binary logistic regression analyses were performed. To manage the effect of the confounding variable during the design and analysis stages, restriction, randomization, standardization, and multivariable analysis were used. The variance inflation factor was used to verify the multicollinearity assumption (VIF < 2.15). To account for potential confounding variables, the variables that demonstrated statistically significant relationships with the dependent variable at *p*-values <0.2 (73, 75, 76) in the bivariable logistic regression analysis were subsequently deemed suitable for a multivariable logistic regression. The Hosmer and Lemeshow goodness-of-fit test was used to check the model’s fitness, and it was well fitted (*p* value >0.05). Finally, a multivariable binary logistic regression model was used to identify variables with a *p*-value <0.05. The degree of association was indicated by an adjusted odds ratio (AOR) with a 95% confidence interval.

## Results

### Socio-demographic characteristics of the study participants

Out of the 633 samples that were chosen, 608 were able to participate, yielding a 96% response rate. Of these, 452 (74.3%) were male and 156 (25.7%) were female. Among these, 248 (40.8%) were married. The majority of participants were orthodox religious followers (556 (91.4%)) and Muslims (52 (8.6%)). The participants’ ages ranged from 21 to 54, with a median age (IQR) of 28. The majority of police officers work in community policing; 308 (50.7%) and 220 (36.2%) of the participants had a college diploma (Table 1).

**Table 1:** Socio-demographic characteristics of police officers working in central Gondar zone, Northwest Ethiopia, 2023 (*n* = 608).

Variable	Category	Frequency (*n*)	Percent
Sex	Male	452	74.3
	Female	156	25.7
Age of participants’	21–29	334	54.9
	30–38	179	29.5
	39–47	65	10.7
	≥48	30	4.9
Religion	Orthodox	556	91.4
	Muslim	52	8.6
Marital status	Single	337	55.4
	Married	248	40.8
	Divorced	23	3.8
Educational level	Grade 10th complete	142	23.4
	Grade 11 and 12 complete	169	27.8
	Diploma	220	36.2
	Bachelor’s degree and above	77	12.6
Work-experience in policing	<5 years	255	41.9
	5–9 years	119	19.6
	10–14 years	87	14.3
	≥15 years	147	24.2
Work place	Urban	250	41.1
	Rural	358	58.9
Children	No	362	59.5
	At least one children	246	40.5
Number of children	1–2	153	25.2
	3–6	93	15.3
Monthly salary	1,825–3,825 ETB	227	37.3
	3,826–4,358 ETB	86	14.2
	4,359–5,646 ETB	160	26.3
	5,647 ETB and above	135	22.2
Organizational rank	Constable	188	30.9
	Sagen	296	48.7
	Inspector	94	15.5
	Commander	30	4.9
Working position	CPC	308	50.7
	PSC	13	2.1
	Administrative staff	195	32.1
	Traffic police	50	8.2
	Crime investigator police	42	6.9

CPC, community policing chief; PSC, police station chief; ETB, Ethiopian birr.

### Behavioral characteristics of the study participants

Of the participants, 155 individuals (25.5%) drank alcohol. The majority (98.7%) of the police officers did not chew khat, and all of them were found to be non-smokers. Only 7.9% of them have work misconduct issues. Of participants, 15.8% use medication for stress relevance, and 434 (71.4%) of participants perform physical exercises (Table 2).

**Table 2:** Behavioral characteristics of police officers working in the central Gondar zone, Northwest Ethiopia, 2023 (*n* = 608).

Variables	Category	Frequency (*n*)	Percent
Alcohol drinking	Yes	155	25.5
	No	453	74.5
Chat chewing	Yes	8	1.3
	No	600	98.7
Medication use	Yes	96	15.8
	No	512	84.2
Physical exercise	Yes	434	71.4
	No	174	28.6
Work misconduct issue	Yes	48	7.9
	No	560	92.1

### Work-related characteristics of police officers working in central Gondar zone, Northwest Ethiopia, 2023 (*n* = 608)

In terms of work-related risk factors, job demand was high for 52.8% of participants; 40.1% had low job recognition; 48.4% had high sleeping troubles; in addition, 14.1% had experienced high levels of organizational stress related to their organization; and 29.4% had high operational stress because of their work duties. Concerning their role at work, 49% of them had low role clarity, and 56.1% had practiced role conflict. In terms of daily working hours, 90% of the police officers worked more than 8 h per day, and 95.5% of them worked both day and night shifts (Table 3).

**Table 3:** Work-related characteristics of police officers in the central Gondar zone, Northwest Ethiopia, 2023 (*n* = 608).

Variable	Category	Frequency (*n*)	Percent
Job demand	Low	287	47.2
	High	321	52.8
Job security	Low	307	50.5
	High	301	49.5
Job recognition	Low	244	40.1
	High	364	59.9
Social support	Low	97	16
	Moderate	374	61.5
	High	137	22.5
Social relations	Poor	231	38
	Good	377	62
Intention to turnover	High	573	94.2
	Low	35	5.8
Job satisfaction	Unsatisfied	79	13.0
	Satisfied	529	87.0
Organizational police stress	Low	224	36.8
	Moderate	298	49.1
	High	86	14.1
Operational police stress	Low	125	20.6
	Moderate	304	50
	High	179	29.4
Daily working hour	≤8 h	61	10.0
	>8 h	547	90.0
Work–family conflicts	No	225	37
	Yes	383	63
Sleeping troubles	Yes	294	48.4
	No	314	51.6
Role conflict	High	341	56.1
	Low	267	43.9
Role clarity	Poor	298	49.0
	Good	310	51.0
Shift work	Morning only	12	2.0
	Evening only	15	2.5
	Moring and evening	581	95.5
Nature of shift work	Fixed	445	73.2
	Irregular	30	4.9
	Rotating	133	21.9

### Response format and scoring method of the instrument for work-related burnout among police officers in central Gondar zone, Northwest Ethiopia, 2023 (*n* = 608)

The response format and scoring method of the instrument are as follows: The items that elicited responses have a five-point Likert scale ranging from very low/never = 1, low/rarely = 2, somewhat/sometimes = 3, high/often = 4, and very high/always = 5. After the data was collected, each Likert scale label was recoded using the following formats: 1 = 0, 2 = 25, 3 = 50, 4 = 75, 5 = 100; for reverse coding, 1 = 5, 2 = 4, 3 = 3, and then all the items were summed. Next, each choice was given one of the following weighted ratings (percentages): never (0%), seldom (25%), sometimes (50%), often (75%), and always (100%) (Table 4).

**Table 4:** Response format and scoring method of the instrument for work-related burnout among police officers in central Gondar zone, Northwest Ethiopia, 2023 (*n* = 608).

Work-related burnout items (Chronbach’s alpha = 0.86)	Response category and scoring					
	Never scoring	Seldom scoring	Sometimes scoring	Often scoring	Always scoring	Score mean (SD)
	0%	25%	50%	75%	100%	
1. Is your work emotionally exhausting?	10.7	16.4	20.9	23.4	28.6	60.69 (33.45)
2. Do you feel burnout because of your work?	13.7	21.5	26.2	18.3	20.4	52.55 (33.09)
3. Does your work frustrate you?	18.8	31.3	24.7	14.1	11.2	41.94 (31.12)
4. Do you feel worn out at the end of the working day?	11.7	32.2	27.3	16	12.8	46.51 (30.05)
5. Are you exhausted in the morning at the thought of another day at work?	33.3	25.5	24.2	9.4	7.2	32.73 (30.74)
6. Do you feel that every working hour is tiring for you?	28.9	33.6	21.1	7.2	9.2	33.55 (30.66)
7. Do you have enough energy for family and friends during leisure time? (Reversed scoring)	3.9	5.9	10.2	24.8	55.1	80.30 (27.41)
Total average score						49.75 (30.93)

### Prevalence of work-related burnout among police officers

According to the study’s findings, 45.7% of police officers in the central Gondar zone had experienced work-related burnout (WRB) in the previous six months [95% CI (41.75, 49.69)]. Table 4 displays the results of all seven work-related burnout items, together with the corresponding frequency and mean scores.

### Factors associated with work-related burnout among police officers

According to the bi-variable logistic regression analysis, WRB was shown to be associated with the following factors: sex, age, place of work, marital status, having children, organizational rank, medication use for stress relevance, exercising, organizational police stress, operational police stress, work-family imbalance, job satisfaction, sleeping troubles, role clarity, job demand, monthly salary, job security, and work experience in policing (*p* < 0.2). However, after adjusting for confounding variables in the multivariable binary logistic regression analysis, the statistically significant variables associated with work-related burnout included participant sex, job demand, job satisfaction, sleeping troubles, and organizational and operational police stress (*p*-value <0.05).

Consequently, individuals with high job demands had a 1.56-fold increased chance of having WRB than did those with low job demands [AOR: 1.561; 95% CI (1.045, 2.332)]. The odds of developing work-related burnout (WRB) were 1.91 times higher among participants who were dissatisfied with their jobs than among those who were satisfied [AOR: 1.910, 95% CI (1.054, 3.461)]. Compared to male participants, female participants had a 4.3-fold increased risk of having WRB [AOR: 4.298; 95% CI (3.274, 8.573)].

The chance of having WRB was 2.23 times greater for those with high organizational police stress than for those with low organizational police stress [AOR: 2.231; 95% CI (1.076, 4.624)]. The risk of developing work-related burnout (WRB) was 2.35 times greater for police officers with moderate operational police stress than for those with low stress [AOR: 2.349; 95% CI (1.309, 4.216)]. The risk of developing WRB was 4.88 times higher for police officers with high operational police stress compared to those with low operational police stress [AOR: 4.881; 95% CI (2.385, 9.992)]. Compared to other participants, those with a high degree of sleep troubles had a 1.52 times higher risk of developing WRB [AOR: 1.522; 95% CI (1.032, 2.244)] (Table 5).

**Table 5:** Bivariable and multivariable binary logistic regression analysis of the factors associated with police officers’ work-related burnout in central Gondar zone, Northwest Ethiopia, 2023 (*n* = 608).

		WRB		COR (95% CI)	AOR (95% CI)
Variables	Categories	Yes	No		
Age	21–29	121	213	0.649 (0.306,1.376)	0.706 (0.257,1.940)
	30–38	107	72	1.698 (0.781,3.694)	1.111 (0.431,2.864)
	39–47	36	29	1.419 (0.595,3.380)	0.700 (0.261,1.877)
	≥48	14	16	1	1
Sex	Male	163	289	1	1
	Female	115	41	4.973 (3.317,7.456)	4.298 (3.274,8.573)**
Marital status	Single	120	217	1	1
	Married	144	104	2.504 (1.788,3.506)	1.298 (0.734,2.296)
	Divorced	14	9	2.813 (1.183,6.691)	1.945 (0.683,5.543)
Place of work	Urban	105	145	1	1
	Rural	173	185	1.291 (0.932,1.788)	0.975 (0.631, 1.507)
Organizational rank	Constable	60	128	0.410 (0.188,0.895)	1.076 (0.358,3.235)
	Sagen	144	152	0.829 (0.391,1.759)	1.442 (0.534,3.896)
	Inspector	58	36	1.410 (0.615,3.230)	1.579 (0.568,4.388)
	Commander	16	14	1	1
Medication use	Yes	50	46	1.354 (0.875,2.095)	1.041 (0.613,1.770)
	No	228	284	1	1
Physical exercise	Yes	170	264	1	1
	No	108	66	2.541 (1.770,3.649)	1.427 (0.898,2.267)
Job demand	Low	103	184	1	1
	High	175	146	2.141 (1.545,2.967)	1.561 (1.045,2.332)*
Organizational police stress	Low	70	154	1	1
	Moderate	147	151	2.142 (1.491,3.077)	1.380 (0.870, 2.190)
	High	61	25	5.368 (3.114,9.253)	2.231 (1.076, 4.624)*
Operational police stress	Low	28	97	1	1
	Moderate	130	174	2.588 (1.605,4.174)	2.349 (1.309, 4.216)*
	High	120	59	7.046 (4.175,11.892)	4.881 (2.385,9.992)**
Work–family conflicts	No	161	222	1	1
	Yes	117	108	1.494 (1.073,2.080)	1.005 (0.670,1.508)
Job satisfaction	Satisfied	227	302	1	1
	Unsatisfied	51	28	2.423 (1.481,3.964)	1.910 (1.054,3.461)*
Sleeping troubles	Yes	161	133	2.038 (1.474,2.819)	1.522 (1.032,2.244)*
	No	117	197	1	1
Role clarity	Low	152	146	1.520 (1.103,2.095)	1.379 (0.916,2.076)
	High	126	184	1	1
Job security	Low	125	182	1	1
	High	153	148	1.505 (1.092,2.074)	1.362 (0.919,2.020)

AOR, Adjusted odds ratio; CI, confidence interval; WRB, work-related burnout; 1, reference category; **statistically significant at *p* < 0.001; *statistically significant at *p* < 0.05.

## Discussion

According to our research, 45.7% (*n* = 278) of the police officers had experienced WRB [95% CI (41.75, 49.69)]. WRB was shown to be significantly associated with sex, job demand, organizational and operational police stress, sleeping troubles, and job satisfaction. This study’s WRB prevalence was nearly identical to that of other studies on police officers in Costa Rica (44.4%) (77) and Mexico (44.16%) (25). This similarity may be explained by the fact that police officers experience burnout in nearly every country worldwide, regardless of their level of development or living conditions (1).

Nonetheless, in comparison with research conducted in Kenya (30.9%) (27), Sri Lanka (31.1%) (52), Mexico (31.1%) (25), Chile (28%) (23), and Spain (32.2%) (21), we found a greater prevalence of WRB. This might be due to variations in the assessment tools used, resource availability such as poor remuneration, poor workplace conditions, high workloads, and low training opportunities (38, 78), unpaid overtime, and the data collection period. During data collection, there was instability, which made police officers more likely to work loaded. In addition, there are not enough technological devices available to minimize their workload, such as computers, security cameras, road traffic lights, and vehicles for emergencies, compared with those available in Spain and Chile (79, 80). Our study result is higher due to sex differences in burnout, and the incorporated variables; both operational and organizational police stress and sleeping troubles may be highly stressful factors in this study. Other possible explanations for such differences might include variations in the research setting, the data collection method, and the variables we are interested in studying, which may affect the results. In this study, a large sample size and appropriate data collection and sampling techniques were used.

In contrast, research conducted in Mexico (54.9%) (48), Brazil (64%) (81), and Nigeria (55.2%) (82) revealed a lower prevalence of work-related burnout. These discrepancies might be attributed to demographic disparities within the research group, for example, the high incidence of violence and assault among military families and the gap in socioeconomic level (83). Compared to their civilian counterparts, military police are more often seen to have difficult employment, and their reduced involvement in organizational decision-making may contribute to their job dissatisfaction and stress (84). Moreover, a high rate of intimate parental violence in military families contributed to a stressful lifestyle brought on by frequent relocation or family separations, which may have a greater negative influence on relationship satisfaction than it does on civilian police. This could result in a serious public health issue that eventually leads to burnout (85). In Mexico, there was a significant increase in road traffic accidents that made traffic police overly demanding and stressful, which caused burnout (86).

This study finding confirmed that, compared to males, women were shown to be more affected by WRB. This result was consistent with studies done in the United States (87), Oregon, southwestern Washington (87), and Nigeria (32). Possible reasons: male and female employees frequently hold disparate opinions about what constitutes stress, where stress comes from, and how to deal with stress when it becomes an issue (88). Compared to men, who are more skilled at problem-focused coping strategies, they are more likely to employ emotional-focused coping techniques (89). Moreover, police organizational culture, such as higher levels of harassment and overt hostility, may cause more female burnout (90). In addition, female officers may experience higher levels of burnout in a work environment marked by feelings of unfairness, sex discrimination, and a lack of friendship (91). Furthermore, they also have a higher likelihood of experiencing emotional exhaustion from their jobs because of their dual responsibilities as policewomen and primary caregivers for their children (32).

Our study’s findings demonstrated a statistical relationship between job demand and WRB. This affirms the fact that there was more job demand during study times in which police officers were required to shoulder the volatile work conditions due to the dissolution of the special police division of law enforcement agencies. This finding was supported by studies in Norway (92), Poland (93), and Kenya (27). This association might be due to the fact that some organizational, social, or physical needs of the work are more difficult to meet; in turn, a lack of resources to balance these demands causes exhaustion and may lead to WRB (94). The other possible reasons were that their work was characterized by long hours, demanding schedules, irregular shift work, frequent night shifts, and significant unpaid overtime, which revealed burnout (8). In addition, these demanding job requirements and responsibilities might lead to more time constraints and less flexibility in how the work is done, which could result in a higher WRB (9). Serious health problems are linked to high work demands among police officers, and having employment resources is a protective factor against WRB (95). Furthermore, it has been frequently noted that officers become fatigued due to long hours and rigorous work schedules, which deplete their energy, compromise their performance, and lead to WRB (96).

In addition, this study demonstrated a strong association between operational police stress and WRB. This finding was in agreement with studies done in Turkey (97) and Nigeria (32). The most likely explanation is that greater levels of occupational stress were associated with higher WRB (98). In addition, a high level of work-related stress can deplete physical and mental resources excessively, which can result in a severe case of WRB (99). The other likely reasons are that police officers who are under stress may not have enough free time to spend with their friends and family, disrupted sleep patterns or low moods that make them exhausted, loss of emotional resources, and exposure to WRB (12). Additional reasons that might be given Operational stresses include public criticism of police behavior, societal perceptions of policing, fear of overuse of force, hostile contact, and work–family conflict, all of which contribute to work-related burnout (WRB) (100).

Moreover, this study revealed a statistically significant association between organizational police stress and WRB. This finding was corroborated with studies in Scotland (101) and Trinidad and Tobago (30). This may be the result of problematic organizational aspects of working as a police officer, such as staff shortages, inadequate resources, time constraints, work overload, excessive administrative tasks, frequent organizational or policy changes, lack of communication among organization members, and staff shortages or inconsistent leadership styles, which are major contributing factors to burnout (7). In addition, organizational stressors, such as insufficient help from superiors and a lack of cooperation with coworkers, are more likely to persist for officers, which may increase the likelihood that they will experience burnout (102).

Furthermore, high sleeping trouble was significantly associated with WRB. This study was supported by studies in North America (44), Sweden (103), Belgium (104), and Mexico (25). Possible reasons: Insufficient rest or irregular sleep patterns (sleep deprivation) may lead to WRB. A troubled circadian rhythm and poor sleep habits may cause fatigue and stress, which are important factors in the development of WRB (105). Furthermore, reduced sleep also impairs cognitive function and may be crucial in the development of WRB (103).

Finally, this study’s results revealed that job satisfaction was significantly associated with WRB. Burnout was more likely to occur among police officers who were dissatisfied with their work. This study result was reconcilable with studies in South Korea (106), Turkey (97), Sri Lanka (52), India (107), and Kenya (27). Possible reason: job satisfaction is used as a buffer against work-related burnout and increases the success of their objectives; in contrast, dissatisfied employees actively search for and come up with new ways but respond passively to their job (108). This is because job satisfaction, which is a positive emotional feeling and a resource, has a significant negative association with WRB. Moreover, job dissatisfaction may result in reduced efficiency and workers’ well-being in general, which in turn causes WRB (97).

### Strengths and limitations of the study

This study is among the few that have examined the prevalence and factors associated with WRB among police officers in Ethiopia. This study used a standardized and reliable tool with a sufficient sample size from several locations, including both urban and rural police departments, and we could extrapolate our results to the whole central Gondar zone police force as well as other similar police organizations in the world. Moreover, this research may contribute to international research as a piece of literature for other researchers intending to work in this area of study and to find pooled prevalence figures of WRB among police officers. However, this finding is limited due to its cross-sectional nature, which cannot rule out the effect of self-reporting bias. Since the data were self-reported prior experiences of police officers, reporting bias and an underestimation of burnout levels may be present. To minimize this bias, we restricted the data to recent experiences only. In addition, it is difficult to generalize the results to other workplaces since it is a cross-sectional study that is specific to police institutions, i.e., police officers. Future studies need to account for a variety of workplaces in order to demonstrate the relationship between various employment features and WRB. With the exception of these restrictions, we believe this study offers reliable evidence to address this work-related burnout among police officers.

## Conclusion

According to this study, a significant number of police officers suffer from burnout due to their work. Police officers’ work-related burnout was found to be predicted by factors such as sex, job demands, job satisfaction, workplace stress, organizational police stress, and sleeping troubles. To address this problem, improving the handling of work pressure, developing a stress management program, finding joy at work by improving interpersonal relationships and working conditions, offering support and inspiration to female police officers, and ensuring sufficient and restful sleep are advised.

## Data availability statement

The original contributions presented in the study are included in the article/supplementary material, further inquiries can be directed to the corresponding author.

## Ethics statement

The studies involving humans were approved by the studies involving human participants were reviewed and approved by Psychological Research and the Institute of Public Health, University of Gondar with an approval number of IPH/2501/2023. The patients/participants provided their written informed consent to participate. The studies were conducted in accordance with the local legislation and institutional requirements. The participants provided their written informed consent to participate in this study.

## Author contributions

AEB: Conceptualization, Data curation, Formal analysis, Funding acquisition, Investigation, Methodology, Project administration, Resources, Software, Supervision, Validation, Visualization, Writing – original draft, Writing – review & editing. DGY: Conceptualization, Data curation, Project administration, Software, Methodology, Supervision, Writing – review & editing. AAB: Conceptualization, Methodology, Software, Supervision, Visualization, Writing – review & editing. ABA: Methodology, Supervision, Writing – review & editing. TA: Methodology, Visualization, Writing – review & editing. CM: Methodology, Software, Writing – review & editing. YMM: Methodology, Visualization, Writing – review & editing. AT: Supervision, Conceptualization, Project administration, Software, Data curation, Methodology, Writing – review & editing.
