# Corrigendum: Prevalence of work-related burnout and associated factors among police officers in central Gondar zone, Northwest Ethiopia, 2023

**DOI:** 10.3389/fpubh.2025.1630242

**Published:** 2025-06-12

**Authors:** Anmut Endalkachew Bezie, Dawit Getachew Yenealem, Azanaw Asega Belay, Alebachew Bitew Abie, Tadiwos Abebaw, Christian Melaku, Yimer Mamaye, Amensisa Hailu Tesfaye

**Affiliations:** ^1^Department of Occupational Health and Safety, College of Medicine and Health Sciences, Wollo University, Dessie, Ethiopia; ^2^Department of Environmental and Occupational Health and Safety, College of Medicine and Health Sciences, Institute of Public Health, University of Gondar, Gondar, Ethiopia; ^3^Department of Environmental Health, College of Medicine and Health Sciences, Wollo University, Dessie, Ethiopia

**Keywords:** Copenhagen Burnout Inventory, work-related burnout, police officer, prevalence, psychosocial risk factors, burnout, Ethiopia

In the published article, there was a typographical error in the **Methods section**: the cut-off values for operational police stress categories were misstated.

A correction has been made to the **Methods section**, *Psychosocial work factors*, Paragraph one. This sentence previously stated:

“According to the operational police stress tool, a score less than 2.0 indicated low stress, a score between 2.1 and 4.6 indicated moderate stress, and a score of 4.7 or higher indicated high stress (51).”

The corrected sentence appears below:

“According to the Operational Police Stress tool, a score ≤ 2.0 indicates low stress, a score between 2.1 and 3.4 indicated moderate stress, and a score of ≥3.5 indicates high stress (51).”

The authors apologize for this error and state that this does not change the scientific conclusions of the article in any way. The original article has been updated.

